# Report of the Committee on Practical Medicine

**Published:** 1860-11

**Authors:** C. Goodbrake

**Affiliations:** Clinton


### REPORT OF THE COMMITTEE ON PRACTICAL MEDICINE.

By C. GOODBRAKE, M. D., of Clinton.

By the Constitution of the Illinois State Medical Society, it is made incumbent upon the Committee of Practical Medicine, to "prepare an annual report on the more important improvements effected in this State in the management of individual diseases; and the progress of epidemics; referring as occasion requires, to medical topography, and to the character of prevailing diseases in special localities, during the term of their service."

Owing to the fact that we have no registration law in our State, which would permit proper data from which we could more easily gather the necessary statistics, it is a very difficult task to comply strictly with the foregoing provision of our Constitution; and in order to overcome, as far as possible, this defect in our State laws, your Committee was compelled to solicit the co-operation of individual members of the profession. Accordingly, soon after the meeting of the Society in Decatur, we sent the following circular to a large number of medical practioners throughout the different sections of the State:

1. What have been the most prevailing diseases in your locality during the year? Give causes, symptoms, treatment, and rate of mortality.

2. Has any unusual epidemics prevailed in your section of country? If so, give character, treatment, etc.

3. Do the ordinary diseases of your region seem to undergo changes from year to year? If so, what are those changes, and what different treatment is necessary?

4. Has Typhoid Fever been prevalent in your section of country? Give your views of the pathology of Typhoid Fever, also, your treatment, etc.

5. Has Cholera Infantum prevailed with you during the year? Give cause, treatment, etc.

6. Has Diphtheria been prevalent with you? If so, give your views of the disease, treatment, etc.

7. Has Stomatitis Materna prevailed in your vicinity? Give suggestions as to causes and treatment.

8. Have you any improvements to suggest in the treatment of any disease?

9. Please give topographical description, so far as practicable, of your locality, together with any facts that may come under your notice, which will be interesting to the profession.

Very few of those to whom the circular was sent, took the trouble to respond; and some who had even promised to aid us in our task, seem proper to forget us entirely. But to those few gentlemen who had the kindness to notice our solicitations, and answer our interogatories, we would take this occasion, in the name of the Society, and in behalf of your Committee in particular, to return our sincere thanks.

Instead of attempting to systematize, in the arrangement of this report, the Chairman has taken the liberty to insert, as near as possible, each individual communication in the order in which they were received; with such comments or remarks as he has deemed necessary or expedient.

The first answer to our circular, was received from Dr. J. N. Niglas of Peoria. He premises his answer to our interogations, with a few very sensible remarks in favor of a registration law.

Dr. N. writes as follows:

"Having duly received your circular, and perused the questions asked, and proposed to the profession in this State, I for one consider it to be a duty to answer, so far as individual experience will permit me to do; and whilst being about to do so, I cannot but complain about the defective condition of our laws and regulations, concerning such matters as are in intimate connection with the advancement of the profession in various ways. Where is there kept any record of the number of births in the different counties? Where of the deaths? How shall a reliable census be arrived at, as to the increase of population, and how is it possible to determine the ratio of mortality in one years time; and either the fatal character of a prevailing disease or the effectiveness of treatment, if no public records are kept; and the physicians day book is the only fountain from which the State Medical Society is to derive information.

The way to answer such interogatories as were asked in your circular, with satisfaction to the Committee and to the Society at large, would be, first to petition for such laws as would enable the secretaries of the different societies in our counties, to obtain correct statistical numbers as to births and burials, with the additional notice of the disease, of which the person had died; also the name and age, and such other remarks as might be deemed necessary. Such is the law in France, Germany, and wherever the government takes interest in medical affairs for the common good of its citizens. And to make it work for the best of your investigations, such a law should be passed by our Legislature, as would command every Sexton, or other persons, throughout the State, not to bury any person without a certificate from the physician last attending the deceased; which certificate he should be compelled to file with the County Clerk.

Thus a firm and reliable basis would be established, and no doubt but the Secretaries of County Societies, though a considerable task this would be to many of them, would in my opinion, cheerfully serve the profession, by forwarding copies of lists or records found in the archives of their several counties.

Permit me sir, to beg your pardon for the premonitory remarks I made, but believe me they are deemed necessary, and are, if reflected upon and carried out, a main and important assistance to every Committee charged with a task as you are now."

Dr. N. replies to the second interogatory in our circular as follows:

"If any disease prevailed during the last twelve months in the city of Peoria and vicinity, it was Scarlet Fever, and it is evident from the preceding part of this communication it passed nearly through all stages an epidemic may possibly pass; and an epidemic I must call it, in as much as in our complex population there are left but very few families in which the disease did not make its appearance. But how it originally came amongst us, whether by contact, or by spontaneous creation of its virulent agent, I cannot be positive; although I am disposed to believe, that we owe it to importation from places in our neighborhood; where, whilst we had Small-pox, this specific fever plagued the inhabitants, as I mentioned before. Certainly there are reasons, which, when taken into consideration, might establish a basis whereupon we possibly could build up a theory for its spontaneous generation amongst us. As early in the spring of the year past, as April and May, diseases commenced to develope, which manifested the character of erysipelas in its various forms and shapes, and though at the outset, they proved to be only a fever characterised by the specific frequency of the pulse, and its specific urine, without any other physical signs; yet, when the temperature was increased, a more decided form was unfolded, as for example: Erysipelas of the face, to which in some cases became accessary, slight symptoms of sore throat, which were followed by the decided appearance of the disease in question. There is no doubt in my mind but that Erysipelas and Scarlet Fever are of a kindred nature, and observations during a number of years, cause me to believe that these two diseases stand to each other in the ratio of an antecedent and its consecutive."

"As to the disease itself, I will now try to give you a description, which, though it is the result of my own individual observations for the last year, or rather to say, since its epidemic appearance among the people of Peoria, may in some way enable you to draw conclusions."

"From all cases which happened to come under my charge, I feel compelled to state that not two cases were alike, in spite of the identity of the disease itself. There were but few cases in which the malady would show its face, as it is commonly depicted by nosographers of the former and the present century. On the contrary, anomalies in every direction seemed to be the rule, and ordinary cases were exceptions. Such anomalies as I allude to, were either in a fragmentary manner uncommonly mild, or severe; so much so, that in some cases I observed the characteristic eruption on the skin, well marked and developed, without any great febrile excitement, or considerable inconvenience from the conjested condition of the capillaries beneath the redened surface, which, in a shorter period than usual turned pale again, followed by desquamation; whilst on the other side there was observable an immense degree of febrile action, accompanied by all and every symptom of severe angina, but without the least mark on the cutis, even the slightest dotted redness of the skin whatever. And such cases which the relatives of my patients were disinclined to believe to be of scarlatinous nature, and Scarlet Fever without its specific exanthem, were proven to be such by decided desquamation, both on the limbs aud trunk of the body, following five or six days from the abatement of fever."

"I also observed but few cases without complications in the various stages of the disease, from the stage of incubation, down to the conclusion by the process of desquamation. Such complications made themselves observable by either functional or organic disorder in the various apparatusses; although at the outset of the disease, when observing disturbances of the brain, either soper or agitation. I feel more disposed to believe that such manifestations ought to be considered exclusively as the consequence of the virulent agent communicated to or taken up into the circulating medium, which on its way through the arterial canals is exerting anomalous irritation, thus causing, according to its more or less poisonous nature, either soper or agitation; whilst, when they are manifested at a later period, I should consider them as indices of an accessory disease to that which was first developed.

Pneumonic and Pleuritic symtoms I meet with very seldom; but more frequently with inflammation of the parotid and sub-maxillary glands, accompanied with swelling of the lymphatic glands on one or both sides of the neck. Angina, though not considered a complication, as it is one of the first symptoms of the whole process, became in few cases the cause of Laryngitis, which in a very short time terminated fatally by membranous exudations—as I am informed by a physician of this city, under whose care the cases occurred, and who had the privilege of a post-mortem in either of the cases. In others diphtheritic exudations, with malignant ulcers on and about the tonsils were observed by myself. From reports gathered up amongst our citizens, I learn that suppuration of the parotid glands and the intermuscular substance of the neck was not unfrequently the fatal close of the scene. From the sudden deaths of a number of children during the year past, I am inclined to suppose that, by either the impaired reflux of blood from the brain, or the vehement action of the heart, and consequently by too copious an accumulation of this liquid in the membranes and substance of the cerebrum, apoplexy and serous effusion in the ventricles took place. At all events, there are not many families in this city who do not deplore one or more victims lost in this epidemic, and there are those who burried from three to four of their children. So much as to the character and rate of mortality."

In regard to the treatment of Scarlet Fever, Dr. N. writes, that in ordinary cases his treatment was *expectative.* He paid strict regard to the diet; allowing no solid food from the beginning. Dilutent drinks were given freely; the patients were kept under a moderate warm covering, by no means heavy; strict attention being paid to the ventilation of the sick-chambers, &c. In cases where the bowels were tardy, they were daily moved by enemata of milk and water, and when the fever was high, by vinegar and water in equal quantities. Only in decided cases of costiveness, castor oil was resorted to.

After giving his reasons for his expectant treatment, he further remarks:

" From the outset I carefully examined whatever patients I

attended, whom I took the privilege of seeing from two to three times a day. The region of the parotid and sub-maxillary glands, which I from the beginning advised to be covered with cotton, and flannel wrapped around the neck; and as soon as the slightest swelling commenced to be perceptible, a liniment composed of Ol. Amygdal. dulc. ℥ ii., Liq. Ammon. Caust. ʒ ii., Ext. Conii. ʒ i., Gum Camphor gr. x, was repeatedly applied during the day, the cotton and flannel wrapped around, and glad I am to say, none of the cases under my charge were lost by consecutive suppuration. In every case, if attended to from the beginning, the swelling would gradually give way without any inconvenience.

" Secondary dropsical affections, when manifested without decided morbid condition of kidnies, I usually treated with dry warm fomentations and sudorfics; yet, when renal affections were observed, simultaneously with edema in the limbs, face, or other parts; or serous exudations in either of the splanchnic cavities, accompanied by a higher degree of sensibility, I applied leeches according to age and the urgency of the symptoms. By way of the mouth, I exhibited calomel with digitalis or acetate of potassa; without, however, forcing the flow of urine by diuretics in a higher degree; except alarming symptoms would command me to exhibit the tinct. canthardies. Milk diet in such cases I think to be very servicable."

To our third interrogatory, Dr. N. makes the following answer:

"As diseases are phenomena produced by certain atmospheric and telluric influences, acting upon animal organization, both in brutes and reasonable beings, which are possessed of individual receptivity, more or less irritability, &c., it cannot but be natural, that, as the productive causes of disorders are to some extent varying in their composition as to the quantitative and qualitative proportions of their components, without effecting their real productiveness of certain diseases, and even specific ones; so the complaints of our race must and will change from time to time, and thus it is; why, on meeting the sick at their bedside and making our medical examination, we sometimes find it very difficult to arrive at a firm and conclusive diagnosis, although there are a number of features observed which seem to be familiar to a disease well known; yet at certain times we are disenabled to accouut for the co-existence of one or the other sign or symptom that were met with. Whether right or wrong I do not know, but I believe we shall not be enabled to give satisfactory reasons for such changes of diseases from time to time, as long as meteorological observations are not made more extensively, accurately and simultaneously in the various sections of this State and the States of the Union. And not only with the thermometer and barometer, but also in regard to the accumulation of the electric fluid, whether it be positive or negative; in what ratio the atmosphere is impregnated with ozone, &c., &c. True, physicians from the name of their professional avocation ought to be familiar with nature's laws, and so they are, at least a majority of the regular sons of Æsculopius now-a-days; yet I do not think that many of our brethren dispersed throughout the different counties of our State, have leisure enough to devote much of their time to observations of this kind; and yet no doubt, in many instances they would lead us to read nature's mystical hieroglyphics when visiting our patients. Much good is done by our medical periodicals, many a new discovery is published for the general good of suffering mankind; yet, none of all I ever saw published among us, took the trouble to devote a few pages in each month to such observations as you will find in foreign Journals.

"What the Smithsonian Institute is gathering by its agents all over the States, might be made to benefit our State Medical Society, and the profession in mass, if each agent was required to send a copy of his report to the editor of one of the Medical Periodicals published in our State.

"To particularize on this topic, would take me too far, as it would be necessary to rehearse the entire index of Pathology, yet so much I will indulge in saying, that in this changeableness of diseases periodically observed, the question among physicians, took its origin, whether a restorative treatment should be generally resorted to, in opposition to antiphlogistic medication. This question, I for one would answer thus:—

The best treatment is that which is best adapted to each individual case and its peculiarities. No general phraseology will do good; two individuals taken with one disease, if treated according to the dictates of a generalizing spirit, may have a very different fate. The one whose constitution claimed an antiphlogistic medication on being subjected to the restorative treatment, may die, whereas, the same under depletory means would have recovered, and vice versa."

From the several communications received in reply to our circular, we condense the following statements:

Dr. R. G. Laughlin, of Heyworth, says that the principal diseases prevalent in that place and vicinity, during the year ending Feb. 20th, 1860, were Pneumonia, Dysentery, Bilious Remittent, and Typhoid Fevers, with a few cases of Diphtheria during the months of November and December.

He thinks that Pneumonia arises from "malarial influences," acting in connection with the "sudden atmospheric changes that usually take place in the months of February, March, and April." Hence, he uses *Quinine* freely in the treatment of the disease, in connection with other remedies, but finds no occasion for Blood-letting.

Dysentery was quite prevalent during the months of August and September, and most of the cases exhibited a tendency to a Typhoid condition. His treatment consisted in the use of Calomel, Opium, Quinine, and the usual astringents, with Norwood's Tict. Veratrum Viride to controll the circulation. He reports no deaths. Bilious Remittent Fevers began to prevail in August, but were promptly amenable to the ordinary remedies. A little later in the season they were more protracted, and showed a tendency to the Typhoid type; and later still Typhoid Fever proper prevailed to a considerable extent. Dr. Laughlin presents nothing new in reference to the treatment of these forms of fever.

Dr. Laughlin met with eight cases of Diphtheria, of whom one died. His treatment was commenced by a mercurial cathartic, followed by alterative doses of mercurials, Opium, and Quinine. Locally, he applied in the more severe cases, a solution of Sulphate of Copper to the throat, and in the milder cases, solutions of Acetate of Lead, Zinc, or Tannin.

In one of his cases certain muscles of the neck became rigidly contracted, and in another, several of those of the neck and extremities became either partially or completely paralyzed, but subsequently recovered. He regards the disease as *Sui Generis.*

Dr. John Wright, of Wapello, communicates an interesting case of Typhoid Fever, which on the 16th day of its progress, exhibited the following symptoms: A desire to go to stool which was not effectual, but was accompanied by a sudden pain in the bowels with a sense of faintness, followed by shivering as in the cold stage of ague. The whole abdomen rapidly became tender to pressure, and more tympanitic than before. The seat of pain and most acute tenderness was below and a little to the right of the umbilicus. The position of the patient was dorsal, with the knees drawn up. Suspecting perforation of the intestine, Dr. W. prescribed half a grain of Morphine, to be repeated every three hours, and hop fomentations to the abdomen. This treatment was continued three days, with some abatement of the pain and tenderness. At the end of the third day the bowels were moved by a dose of Castor Oil and Oil of Turpentine, and followed by Morphine, half a grain every two hours until the patient slept. Under this influence, aided by a pill of Nitrate of Silver and Opium, the patient slowly improved for six days; when the sub-maxillary and parotid glands of the right side began rapidly to swell; and soon rendered both respiration and deglutition difficult. Half a grain of calomel was now added to the Morphine, and a poultice applied to the swelled glands. The glands suppurated, and the pus was discharged on the fifth day. The difficulty of deglutition, however, continued, and the patient gradually failed, and died 24 days after the glands began to swell, and 33 days after the sudden attack of pain in the abdomen. A *Post Mortem* examination revealed an adhesion of the peritoneium covering a portion of the ilium to the abdominal walls, and on separating this adhesion, the intestine was found *perforated*, and many ulcerations in its mucous membrane.

The following report from J. W. Coleman, M. D., of Le-Roy, McLean Co., we copy entire:

*Report of an Epidemic of Dysentery that prevailed at Le-Roy during the summer and autumn of* 1858.

This disease made its appearance in our community about the first of August. The first few cases were of a mild form, and far the most part among children, and yielded readily to treatment. After a week had elapsed it began to assume a more malignant character, and all suffered alike—no class, age, or sex was exempt.

There was nothing peculiar in the epidemic at its beginning. The attacks were sometimes ushered in with a chill, followed by fever of an intermittent or remittent grade, and sometimes with a Diarrhœa, and in a few instances with Cholera Morbus. The cases were attended with bilious symptoms; tongue was covered with a white or yellow fur; nausea and vomiting were of frequent occurrence; urine scanty and high colored; pulse full and frequent in some, and in others small and irregular; want of appetite and anxiety of countenance.

Some of the patients for several days before they were taken down, complained of uneasiness and pain in the bowels, with occasional dysenteric discharges, want of appetite, furred tongue, weakness and trembling, when suddenly they would be taken down with copious stools composed to all appearances of decomposed blood, shreds of mucous membrane, vitiated bile, rice water, and a small quantity of granulated fœces, and would sink rapidly under them, or pass into a collapse resembling cholera.

We here give a case of Mrs. D.———, age thirty-two years, mother of four children, bilious temperment. Previous to our visit she had been complaining of pain, with an occasional discharge of blood and mucus, but was not sufficiently sick to keep her bed; we prescribed the usual remedies without much apparent benefit. On the morning of September 6th, we received a hasty summons to visit the lady. When we reached the patient, we found that she had had, as the friends termed it, a terrible run on her bowels during the night, the discharges numbered from fifteen to twenty during the past twelve hours of the consistance above mentioned, and very large in quantity, at times the night vessel was two-thirds full.

Her condition in the morning was sorry enough. She believed, and we had but little reason to doubt it, that death would soon end her suffering. Her countenance was livid and anxious, eyes sunken, frequent sighing, great restlessness, face and body bedewed with a cold clammy sweet, pulse small and irregular, thirst great, extremities cold and the discharges returning at intervals of every twenty minutes or half hour.

We promptly put her on a sustaining treatment. Had heated flannels and bricks put to her extremities, gave beef tea well salted, ad-libitum, brandy and quinine as a stimulant tonic, and to controll the frequency of the discharges :

℞ Pulv. Opii. 11 gr.
Acetas Plumbi. iv. gr.

Every four hours in vinegar, alternated with the Turpentine Emulsion. Under this treatment the vital powers rallied within the next twenty-four hours, and the case progressed as an ordinary case of Dysentery, though it was slow and lingering, and the patient was long in getting up.

In all cases there were more or less fever, mostly of an intermittent or remittent character, and quinine in every stage of the disease was used with benefit. Among small children there was considerable cerebral disturbance, and towards the termination of fatal cases, convulsions often were present.

*Topography and Cause.*

Those of us physicians practising in our village, thought the prevalence of the disease was owing to the wet spring followed by sultry weather, generating malaria. The Spring, as all the gentlemen of the Society remember, was exceedingly wet. Raining almost the exact time that preceded the deluge, forty days and forty nights. For some six weeks the whole earth in this vicinity was thoroughly saturated.

Dry weather commenced about the first of June, and it continued dry and warm until the middle of August, when the most intense hot weather set in that was ever known in central Illinois. For days and days the mercury in the Thermometer stood at about the same figure. People in the streets and fields seemed to absolutely wilt beneath the rays of the sun.

First of August brought us Dysentery. That malaria had much to do with it, or indeed was the prime cause, we may conclude from most all the cases of fever, and they were many, both at the time and subsequently put on the intermittent or remittent grade. We even believe that we can trace the Topography of it so close as to see that it almost wholly confined itself to localities where malaria was most rife. Thus, in the locality where it first made its appearance, and where most cases occured, it was particularly the case. In our village the most cases and most number of deaths occurred in a portion or street that was comprehended between two prairie sloughs, forming a sort of triangle. The sloughs passed by two old steam mills, which from the situation and condition could not fail to generate malaria. There could not have been less than thirty cases, and six deaths. In the north half of the town, there were several cases, but they bear no comparison with the number on this one individual street.

*Another Locality.*—On the Bloomington road, five miles from Le-Roy, there is a tract of land of eighty or one hundred acres in extent, which was a total pond, and water stood on it, and rank vegetation grew the whole summer season; around this there was as many as twenty patients, and five or six deaths.

For some miles along the north side of "Old Town Timber," and about the site of the "Old Indian Village," many cases occurred, and in most every instance the locality was one where sloughs were near or the prairie low and wet.

While it prevailed so greatly near wet places—all those settlements on the high and rolling prairie were in a manner exempt. Thus Merrifield's ridge, the past prairie, the prairie lying between Le-Roy and Randoph's Grove, and the prairie north of us, lying between our village and Old Town Timber.

*Mortality.*

The percentage of deaths to the number of cases, not keeping notes, we cannot come to a very near approximation, but we would think not more than one in fifteen.

*Duration.*

The duration of the disease was from a few days to six weeks; depending in a great measure on the management of the first or forming stages of the disease. In cases where a physician had been neglected to be called in, or improperly treated, the patients either sank from exhaustion, being worn out by tormina and tenesmus, and the waste of system from discharges, or the cases assumed a continued form, calling for a protracted and wearisome course of treatment.

Several of this class of cases at old Town Timber, came into the hands of Dr. S. W. Noble; two of whom died in four or five weeks of the disease respectively: the first from ulceration of the coats of the intestines, and the second from hemorrhage.

In the treatment of these cases, the physician who preceded him placed his main reliance on Dover's Powders and Tannic Acid. As might be expected, his patients either died or fell into other hands for treatment.

*Treatment,*

When early applied for, was generally successful. But it was a matter of frequent remark among us, that never was there an epidemic of any kind in this region of country that required more discrimination on the part of the practitioner in selecting and administering his remedies, than this one.

Most of the cases required mercurials at the start, combined with opium, and if there was any periodicity in the accompanying fever, we used quinine freely.

The indications of cure were evidently to relieve pain, restore the secretions, and prevent a recurrence of the symptoms. We generally commenced the treatment with something like this:—

| | | |
|---|---|---|
| ℞. | Sub. Murias Hydrarg., | gr. xii. |
| | Pulv. Opii., | gr. x. |
| | Carb. Soda, | gr. xxx. |

Mix, and divide into six powders, one to be taken every three or four hours, according to the severity of the symptoms, and continued them until bilious discharges were gotten up.

The opium was continued with an open hand, not being governed by any fixed rules in its administration. If one, two, or three grains, did not produce the desired effect, we gave the fourth. In many cases it was given until narcotism was produced.

For those cases, and they formed the large majority met with, similar to the one mentioned early in the report, where the discharges were so large and exhausing, we prescribed—

℞. Pulv. Opii, gr. x.
Plumbi Acetas, gr. xxx.

Mix, and divide into six powders, one to be taken every four hours, alternating with a teaspoonful of the following:—

℞. Tincture Camphor,

Tincture Kino,

Paregoric, } aa.

This mixture, it is due to say, was first used by my friend Dr. Noble, and was found very useful in our hands during the whole season that the disease prevailed.

Quite early in the epidemic it was found that opium in the form of tincture, was more liable to produce its constitutional effect, than in the gum or pulverized, and when combined with tincture camphor, equal parts, it had a still better effect in relieving the tormina and tenesmus. No specific effect is claimed for the camphor, but its efficiency is attributed to its well known anti-spasmodic action.

It is not necessary for us to enter any further into the details of treatment. Injections of cold water, starch and laudanum, nitrate of silver, pills of opium and nitrate of silver, the salines, &c., were used, but nothing seemed to answer the indications as well as the treatment described.

Dr. J. O. Harris, of Ottawa, sends us the following:—

"Although I have not been able to attend the meetings of the Society, I am much interested in its success and welfare; and believe that all *true* medical men are in duty bound to contribute something, be it ever so little, to the medical literature of the State. Believing this, I regret that I have postponed writing till this late day, as I can have no time to write at length upon the topics suggested in your circular.

"With regard to your queries, 2nd, 5th, 6th and 7th, I answer briefly, no. Although there have been some few cases of cholera infantum, and during the summer months, diarrhœa among children was extremely prevalent. In this latter disease I found that after exhausting all the usual remedies advised by our standard authors and by my brother physicians, that quinine in *full* doses, frequently repeated, acted (or seemed to act) admirably. I thought at the time that I was prescribing empirically, and now I do not pretend to explain the modus operandi of the remedy—I only know this, that my patients recovered under the use of quinine, and I still frequently prescribe it when I see no particular indication for its use.

"I have never been a hobbyist with regard to quinine—ordinarily I have a clear conception of what I wish to do, and what accomplish with a particular remedy, before I prescribe; but I am free to confess that this drug may be required—and not unfrequently either—when I cannot see precisely *how* it is to act."

In regard to typhoid fever, the Dr. writes as follows:—

"The typhoid fever question is a vexed one with us in Ottawa. Some are almost constantly prescribing for a disease which they call by that name; while others—myself among the number—*very rarely* meet with that disease.

"I have practised here between seven and eight years—I have had patients in all parts of the town, and in every direction in the country, *and have not had more than five cases* which I could call typhoid fever. You say—'Give your views on the pathology of the disease'; it is unnecessary, as I take Wood and Watson for authority, and in all essential particulars accept their opinions. Our 'typhoid doctors' aver that they do the same; yet I notice that their patients have this disease every year or two, and sometimes twice during the same year; that, (taking the physicians' statements) they do not have the *Rose Spots*—often no looseness of the bowels, and that they almost invariably recover in from 5 to 15 days! Taking all these (and more else) into consideration, I am forced to believe, that with regard to typhoid fever prevailing here, it is all *humbug.* Even the unprofessional see through the flimsy pretext, as I heard a lady remark the other day, that when she was taken sick again, she would send for Dr. Blank, so that she could have a spell of the typhoid fever!

"Now, I beg you will do me the justice to believe, that in writing as I have, it has not been to gratify any private feeling—I am on good terms with these typhoid fever doctors—I could not write less and give you my opinion.

"I have tried to induce some of these gentlemen to give you their views upon these questions; but as they seldom write for our local Societies, it is not probable that you will hear from them.

"I believe that meteorological changes have a decided influence upon prevailing diseases; but as yet, I have found no extensive theories with regard to this subject. I should, however, be very glad if the Society would advise its members to keep records like that accompanying this communication. The whole could then be placed in the hands of some competent man for reduction, and some important facts would undoubtedly be eliminated. If this should be done, the hours of observation should be uniform, say at 7 A. M., and at 2 and 9 P. M., and Green's thermometers should be used, as most of those in common use are not reliable.

"I wish the Society would take some steps to secure the passage of an act, giving us the unclaimed bodies of paupers and criminals for dissection. I have already made some efforts in that direction; but as yet, without any good result."

### METEOROLOGICAL RECORD,

*Kept at Ottawa, Ill., by J. O. Harris, M. D.*

| MONTHS. 1859. | Maximum Temperature. | Minimum Temperature. | Max. Mean Temperature. | Min. Mean Temperature. | Monthly Me'n Temperature. | Number of Snow Storms. | Inches Snow. | Number Rain Storms. | Inches Rain & Molten Snow. | DISEASES TREATED. |
|---|---|---|---|---|---|---|---|---|---|---|
| January... | 47 | -15 | 39.33 | --1 33 | 24.89 | 3 | 3.00 | 4 | 1.713 | Rheumatism, Pneumonia, Remittent Fevers. |
| February.. | 67 | 2 | 54.00 | 6.00 | 28.92 | 4 | 8.00 | 4 | 0.860 | Colds, Pneumonia, Remit. Fevers. |
| March.... | 69 | 23 | 57.00 | 31.66 | 40.74 | 3 | 2.75 | 8 | 5.243 | Mam'ry Absces's,* Pneumonia |
| April..... | 70 | 28 | 61.33 | 27.33 | 45.04 | 1 | 0.25 | 10 | 4.082 | Remit. Fevers, Pneumonia, Phthisics. |
| May...... | 81 | 41 | 74.00 | 51.66 | 62.95 | .... | ...... | 13 | 3.120 | Intermit Fevers, Neuralgia, Epilepsy. |
| June...... | 91 | 51 | 84.33 | 48.00 | 65.81 | .... | ...... | 13 | 1.690 | Remit. and Intermit. Fev'rs, Diarrhœa. |
| July...... | 96 | 45 | 86.66 | 54.00 | 75.23 | .... | ...... | 8 | 0.732 | Do. Scarlitina do. |
| August.... | 91 | 50 | 79.66 | 66.00 | 72.13 | .... | ...... | 7 | 3.440 | Do. Diarrhœa. |
| September | 87 | 44 | 75.33 | 51.66 | 61.10 | .... | ...... | 10 | 1.563 | Do. do. |
| October... | 79 | 21 | 65.66 | 31.33 | 47.59 | .... | ...... | 5 | 2.342 | Do. Epilepsy, Dropsy. |
| November | 70 | 12 | 63.00 | 17.66 | 39.53 | 1 | 0.75 | 7 | 2.089 | Do. Colds, do. |
| December. | 41 | -20 | 33.00 | -15.00 | 18.06 | 5 | 8.75 | 4 | 0.944 | Remit. Fev., do. do. |
| TOTALS.......................... | | | | | | 17 | 23.50 | 88 | 27.818 | * These were very frequent about this time—never saw so many cases during the same length of time before. |

Annual mean temperature, 48-49° ; Height of station above the Sea, 500 feet ; Hours of Observation, 7 A. M. and 9 P. M.

"I presume there were other diseases prevailing to some extent—I have only given some of the principal ones that I treated."

As Dr. Harris remarks,—" The typhoid fever question is a vexed one." After all that has been said and written on the subject, it is very obvious that there is still a great diversity of opinion among the members of our profession throughout the State in regard to this disease. I believe that this difference of opinion arises from the fact that most of our physicians fall in with the idea that typhoid fever is a specific disease, and when they come to apply this theory in their practice, they cannot make it correspond with what meets their observation. The mistake—if it be one—has its origin with our standard authorities. From them our medical men get the opinion that the disease is a specific one—as much so as small-pox, scarlet fever, or measles. It is assumed, that in order to make out a case of typhoid fever, the patient must have been getting sick a certain number of days; he must then get worse and send for his physician; the Doctor informs him that he must expect to be sick from six to eight weeks; that he must not expect medicine to do him much good; and that if he gets well he may thank Providence and his good constitution for carrying him through! During the course of the disease, the medical attendant expects to find the tongue furred with red edges; rose-spots, sudamina, tympanitis, diarrhœa, etc., etc.; and if the patient dies and a post-mortem examination is allowed, he expects to find Peyer's glands affected, and suspects strongly that there must be perforation of the intestines; and if he fails to find all these symptoms and lesions, he is led by his authorities to doubt its having been a case of typhoid fever. Cases may occur, where all these symptoms supervene at some period throughout the progress of the disease—and on the other hand I have not the least doubt but that there are cases of the disease under consideration where more or less of these symptoms are wanting; and in just such cases physicians differ as to the name of the disease.

I believe, too, that a person may have an attack of intermittent fever; this, by mismanagement, or otherwise, may become remittent, and may finally terminate in a genuine case of typhoid fever. I treated several such cases during last summer, and the same kind of cases treated by others who contend that typhoid fever is a specific disease, and they would tell me that theirs were not gradually becoming typhoid, but that they were "*assuming a typhoid condition.*"

If typhoid fever is a specific disease, it must originate from a specific cause. And yet, I believe that four persons may be subjected to the same cause of disease; one of them will perhaps escape with a sore throat; the second may have an attack of fever and ague; the third probably remittent fever; and the fourth may become the subject of genuine typhoid fever. And I would account for this, by their system being in different conditions, when exposed to the common cause. But neither of them will have small pox or measles.

Neither do I believe that typhoid fever must necessarily run a certain course, and that it cannot by any possible treatment be cut short. I have had patients who I verily believe had typhoid fever, get well at all periods in from five or six days to as many weeks. It might be argued that I mistook the disease,—and it may be that I did—as I make no pretensions to infallibility in distinguishing diseases—although I will presume to say that I have paid considerable attention to the pathology of the disease under consideration during the last ten years. But be this as it may, I know that I have seen Prof. N. S. Davis cure cases (at the Mercy Hospital, Chicago,) which he pronounced typhoid fever, in from a few days to so many weeks; and I will accept Dr. Davis' diagnosis with as great faith, as that of any man in this or any other country. My candid opinion is, that if we were to avoid names ot diseases as much as possible, and treat diseases according to their symptoms, it would redound to the benefit of our patients; and the differences of opinion among the members of our profession would be thereby greatly lessened.

### NOTES

CONTRIBUTED BY DR. PRINCE, OF JACKSONVILLE, ILL.

### *Diphtheria.*

During the winter months pseudo membranous inflammation of the respiratory passages was prevalent. Sometimes this inflammation predominently affected the larynx, giving rise to croup; and at other times a bronchitis predominated over the laryngeal and tracheal inflammation, though in fatal cases these were very commonly combined. At other times the disease was confined to the nostrils, pharynx, and mouth, sometimes independent of any cutaneous eruption; and at other times accompanying or following scarlet fever, which was at the same time epidemic, so as to lead to the suspicion that this diphtheria is one and the same disease with scarlet fever, only assuming a different form. The disease manifested a tendency to travel or spread from its point of origin. This origin has more often been the palate and fauces. In one fatal case which I saw under the treatment of another practitioner, the larynx escaped until within a few hours of death, when croupy respiration manifested itself.

An epidemic of dysentery may occur with such a history as to lead us to believe that it is contagious; but we do not immediately give the disease a new name. It is not difficult to suppose that an epidemic of dysentery or of cynanche, may be contagious in one period of its duration, and not in another; in one immediate locality, and not in another, owing to the greater or less perversion of the secretions. I have just been reading in the April number of the *Chicago Med. Examiner* an excellent article upon the subject of diphtheria, by Dr. Wm. L. Wells, of Milwaukee, in which it is abundantly shown, that this is no new disease, but only a new naming and classification of a form of disease known from the earliest times of medical record.

In this case there was no distinct false membrane, but a sanious discharge from the mucous membranes—especially from the nostrils. The discrasy seemed too great for the exudations or secretions to consolidate to much extent. The little consolidation which did take place, was in minute specks or scales, soon loosened and displaced by the fluid around them. I cannot add any thing to the descriptiou so often given of this disease. It was universally attended with prostration, indicating the necessity for a supporting treatment.

The fact of the contagiousness of this disease is made a diagnostic between this and other diseases; but this is a distinction which can only be recognised in the progress of an epidemic, and it is therefore of very little diagnostic value.

The local application of strong solution of nitrate of silver, has not acted as satisfactorily as I had been prepared to expect. A weak infusion of capsicum and a saturated solution of chlorate of potash, singly or in combination, have proved far more satisfactory. In the only cases in which I have seen the muriated tincture of iron applied (in the hands of another practitioner,) its effect was not such as to encourage me to use it.

The cautious use of alcoholic stimulants, the liberal use of concentrated nourishment, and the laxatives which are least antiphlogistic, have seemed to be the most rational as well as the most successful treatment.

---

*Tracheotomy in the Diphtheritic Laryngeal Inflammation.*

If the name croup is to be confined to the designation of those cases of false membranous inflammation of the larynx arising and remaining in this organ, and not traveling out of it: the operation of tracheotomy ought to be nearly as successful in croup, as when performed for the elimination of foreign bodies.

Assuming from the absence of ashy tonsils or other signs of false membranous consolidation upon the mucous membrane above the larynx, and from the absence of the mucous rattle in the chest, that the case is one of uncomplicated croup: the operation in such a case ought to afford rest to the swollen vocal cords, and give the patient abundant breath and almost an assurance of a safe termination.

Yet, it is safe to say, that in a great majority of these cases, the mucous rattle will soon be developed after the patient has seemed to have been saved by the operation, and the hopes so fondly cherished by friends and medical attendants, will be disappointed.

I have operated in five such cases, and have not had the good fortune to save one of them. In three of the cases, death was immediate, and there was no such reaction as to inspire hope after the operation. In the other two, death seemed to be equally impending; but after the opening into the trachea, such was the relief from all distressing and alarming symptoms, as to lead to the expectation of recovery. In both of these cases, death resulted in about four days from the operation from mucous inflammation within the chest. In one of these cases Solution of Nitrate of Silver was applied to the trachea, and in the other not. Were I again to operate, I should make a very free application of this or other mild caustics, (oresthetics in Tully's classification.)

Were it not for the horror of the operation, it would be resorted to in a greater number of the severe cases of laryngeal inflammation, for when a patient is cyanosed from a deficiency of oxygen in his blood, the occurrence of death soon after this or any other remedy is resorted to, is not likely to be attributed to the remedy itself. In one of the two cases which I have mentioned, the anaesthesia from deficient elimination of carbonic acid was so great, that the child scarcely felt the operation, and yet, after a few hours' sleep subsequent to the operation, he was able to sit up and amuse himself with his playthings.

From the blueness of the skin in deep anaesthesia from ether and chloroform, we are justified in supposing that the amount of elimination of carbonic acid is diminished. If this is so, the employment of the agents to procure insensibility, may not be altogether safe, and in two instances in which I procured insensibility by the inhalation of a mixture of ether and chloroform, I suspected that the inhalation had increased the previous cyanosis.

I have never practised catheterism of the larynx and trachea after the manner of Horace Greene.

---

*Scarlet Fever, and Typhoid Fever.—Use of Veratrum Viride.*

The number of deaths occuring during the early portion of the winter from Scarlet Fever, was such as to create a public panic, and yet in some families the cases were all very mild, and other families escaped altogether. This was coincident with the prevalence of cynanche maligna, (Diphtheria,) while Typhoid Fever prevailed to an extent almost to make it epidemic. These three coincident epidemics disappeared, as they had commenced, nearly at the same time.

The mild cases seemed to yield readily to treatment whether homeopathic or by the use of appreciable doses, while the severe cases were well calculated to generate scepticism with regard to the power of treatment to control the progress of the disease.

Some of the cases of scarlatina manifested much excitement in the first stage, and in two cases I used Norwood's Tincture Veratrum Viride. This acted like a charm in controlling excitement, and moderating the frequency of the pulse. The remedy has seemed to me to be a powerful antiphlogistic, requiring to be accompanied or followed by stimulants in diseases tending to terminate in adynamic conditions.

The necessity for the use of stimulants in the progress of disease is, however, very far from being a condemnation of the previous use of depressing agents in the former period of the disease. We all know that in injuries and operations involving the certainty of inflammation of the membranes of the brain, the only safety is in depletion to an extreme degree for the first few days; and if in the progress of the case we have to nourish and stimulate freely, we do not call this a reflection upon the correctness of the preceding treatment. We well enough know that if the treatment upon which the patient gets well, should be employed in the earlier periods of the treatment, death would be the inevitable result.

In diseases we may expect an earlier abatement of the sthenic symptoms than in the example adduced, and it is very necessary not to comfound the expression of adynamic congestion with that of sthenic inflammation.

In one of these cases in which the Veratrum Viride was used, the patient died with blue skin, indicating internal adynamic congestion. The other recovered after the free use of alcoholic stimulants.

With my present experience, I should use the stimulating treatment more speedily after the control of the pulse by means of the Veratrum Viride, and employ it very freely.

We are in danger of falling into the error of over stimulating, without previous *elimination* of the retained secretions of sluggish glands; now that the medical world is all in a rage to stimulate, while we may regard a case under treatment as one to require supporting treatment during its progress; we may commit a fatal error, if we fail to administer previously or coincidently a cleaning out dose, which shall make the patient conscious that he has had bile in him.

I have never given veratrum viride in but one case of typhoid fever. In this case a pulse of 140 was readily reduced to 80 by four drop doses, repeated every four hours as long as might be necessary, and renewed upon the rising of the pulse and repeated as soon as found necessary. Notwithstanding some nausea and vomiting, the patient seemed to derive positive comfort from the remedy. Its occasional exhibition was kept up during several successive days. The patient recovered after a long course, using concentrated nourishment and alcoholic stimulants pretty freely at last.

Mercurial cathartics were frequently given during the course of this case with apparent advantage. These were given to remove supposed glandular congestions, and not with any view of their being otherwise curative.

I have been led from this and the majority of the cases of typhoid fever which have fallen under my observation, to doubt the existence of any enteritis as a necessary accompaniment of the disease. That inflammation is frequently present is not denied. It is suspected that the inflammation is only a frequent and accidental development, in an idiopathic disease.

The tympanitic condition is very far from being any evidence of inflammation. It is often a symptom of hysteria from gas within the intestinal canal, readily passing off under the stimulus of aloes and asafœtida. In one of the most prostrated cases of typhoid fever which has fallen under my notice during the past season, there was an enormous development of gas within the alimentary canal, which would accumulate, producing extreme frequency of pulse and shortness of breath; but on introducing a tube into the rectum and allowing it to pass off, the pulse would fall in frequency and the respiration increase in depth. Previous to the evacuation of the gas, there would seem to be tenderness on pressure upon the abdomen; but afterwards, no tenderness at all. Many of the cases of sunken abdomen with tenderness on pressure, are examples of true mucous inflammation, requiring the liberal use of opium and the cautious employment of laxatives.

How important it is to treat diseases, not according to names, but according to conditions.

The following communication, from the pen of Dr. Nance, we also insert entire:—

LA FAYETTE, Stark Co., *April* 23, 1860.

C. GOODBRAKE, M. D.,

*Dear Sir:*—The Circular issued by the Committee on Practical Medicine, of whom you are President, was duly received, and should have been responded ere this; but business has prevented it. I humbly hope, even yet, my meagre contribution may meet with a welcome from my professional brethren, and especially from the President of the Committee on Practical Medicine.

I shall endeavor to answer the interrogatories propounded by your committee in as brief a manner as possible. And in reply to No. 1, "What have been the most prevailing diseases in your locality during the year?" I would answer that the year 1859 and up to the present in 1860, has been one peculiarly favored by unusual health. I would say that I have been engaged in the profession in this place for nearly fifteen years, and am confident that no year during this time has been so healthy. The question propounded is one hard to answer; it would be extremely difficult to select any one disease that has prevailed very generally, and certainly none as an epidemic, until within a month or two in 1860; during which time epidemic Erysipelas has prevailed.

During the latter months of summer and early months of fall, we always have more or less of intermittent fever on the streams, and occasionally on the prairies; but since our country has become almost one *dense* farm, and no fresh turf in our prairies is being turned over, this disease is disappearing from us with great rapidity; and would say to the eastern family who contemplated emigrating to our fair land, that they need fear the ague no more in Illinois on our high prairies. As those miasmatic diseases of former years are fast disappearing from amongst us, it would be supposed *apriori* that their places would be supplied by some other diseases; and I think it can safely be said, typhoid fever has become a more common disease; so has phthisis pulmonalis, and I need hardly remark that scarlatina has become much more frequent, and its type of latter years has usually been very malignant. I think I practised medicine for six or eight years before I even witnessed a case of genuine pseudo membranous croup. Never saw a case of granular disease of the kidneys until the year 1856, since which time I have seen quite a number.

In regard to the ratio of mortality, I would say that it is certainly greater than of former years to the number of patients. All physicians regard intermittent and remittent fevers as the simplest types of febrile diseases, and of course would expect to find the mortality less than in more grave diseases; and this is certainly the case. When we had scarcely any other diseases to treat but these, in the summer and fall, we lost but very few patients, and consequently the responsibilities resting upon us were not great; hence, it was a pleasure to practise medicine, as nearly all our patients recovered. But as the diseases of our country have changed so much, and as we have such a great variety to treat, and the type is so very different, we are in a continued state of suspense and uneasiness when treating most of the diseases that present themselves to us. I think those remarks not only apply to this immediate locality, but will be pretty freely concurred in by all medical men residing north of 40° in this State, as our State north of this degree has settled up and been improved about the same time.

No epidemic prevailed in this vicinity in the year 1859. Since March first in this year, erysipelas has prevailed to some extent. I have seen during this time seven patients; in every instance it made its appearance on the face, usually on the side of the nose; one instance on the lips, one on the brow, and one originated from the boring or puncturing of the ear in a young lady of some 26 or 28 years old; she was sadly paid for her *fashionable* trouble, as it came near causing her death. Of the seven patients under my care, one died—the patient was a lady of 47 years, nervo-lymphatic temperament, rather plethoric, but relaxed fibre. This was the third attack she had suffered from in three different years—connected with the erysipelas she always had derangement of the liver and bowels, amounting to great torpor of the portal circle, constipation and bilious cholic—also fever of an intermittent type. Gave her calomel followed by spts. turpentine, and oil ricini followed with sul. quinine and muriated tinct. ferri, to be continued *per re nata*—morphia sulphas with Doveri sufficient to keep the system quiet—tinct. iodine, to be freely applied over the part affected. I would remark that the erysipelas did not make its appearance until the fourth day from the attack; regarded the case as one of congestion of the lungs, liver and bowels; think she would have died had not the erysipelas made its appearance at all. I treated all my patients pretty much on the same principles, and with success; some cases I applied collodion instead of tinct. iodine—cannot say that iodine has any advantage over collodion; of the two, think I should prefer the latter in most cases.

In regard to our diseases during the past winter and present spring, I would say they have been very different from usual during the same time in former years. After the *customary* January thaw, pneumonia usually makes its appearance, also catarrhal diseases, especially amongst infants, and occasionally amongst adults—sometimes pleuritis or pleuro-pneumonia.—But this winter and spring, none of those diseases have prevailed to any extent. I have not seen more than three or four patients with pneumonia; not more than five or six with catarrhal fever, and none with pleuritis. Instead of sthenic or asthenic inflammatory diseases of the respiratory organs, as is usual during the latter part of winter and first few weeks in spring, our diseases have been congestive in their character, and instead of the disease as is usual being located upon those organs, the congestion has been principally confined to the liver and bowels ; in some cases general congestion seemed to be the type, affecting the brain and lungs, and also extending entirely through the whole chylopoietic viscera. But in my practice the congestion has been principally confined to the bowels, producing general constipation, cholic, uneasy sensation throughout this region, and in a few instances producing mucous and sanguineous discharges, resembling well formed dysentery. In no case have I seen active inflammation in the bowels, with the exception of two cases of puerperal peritonitis. I treated those congestion cases by the free administration of prot. chlo. hyd., combined with pulv. rhei, given in doses of 4 or 5 grs. of the former with 8 or 10 of the latter, given every five hours until the bowels moved; and when I found a patient who could not take the rhei, I gave oil ricini four or five hours after the administration of the mercurials. Sometimes I found it very difficult to move the bowels, then enemata were ordered. On inspection of the alvine evacuations, they were invariably found of a dark hue, usually quite green. The continuation of this course of mild purgation invariably brought about a healthy appearance of the stools; after which convalescence was established. I would remark, that during the interval that purgatives were not given, I ordered sulphas quinia every two or three hours; and in some cases, it was found indispensible to administer some form of an opiate to relieve the severe cholic that some suffered under. Patients did not convalesce as rapidly as after diseases of the lungs of a sthenic type. Some cases were quite protracted, or rather changed into a chronic diarrhœa; this was treated quite successfully by the turpentine and tinct. opium emulsion

*Ques. No.* 4.—Typhoid fever has prevailed to some extent —in one family seven patients had it. I hold that it is strictly an *enteric* disease, and is not susceptible of being but very little abreviated by any treatment;—three of the patients in this family had hemorrhagic discharges; two had petechia, and all had sudamina; all had diarrhœa and tympanitis. All of those patients recovered with the exception of one, who was an invalid before she was attacked; had been suffering for years from hepatitis. I diagnosed softening of the liver previous to death. Several other cases of typhoid fever came under my care during the fall; but in no other instance was there more than two had it in the same house.

My treatment, stated in brief, consisted of spts. nitri. dulc ℥ viii; Norwood's tinct. veratrum viride gtts. xl; mix, give one teaspoonful to an adult every three hours so long as the active stage remains, increasing or diminishing the dose as the case requires. It may be necessary to continue this medicine for eight or ten days or more. I also prescribe at the same time turpentine emulsion, usually combined with tinct. opii: the first prescription subdued the activity of the pulse, and acts favorably upon the urinary and perspiratory systems; the latter containing laudanum, quiets the nerves, promotes sleep, and the spts. turpentine has its specific action upon the glands of the mucous membrane of the bowels. I use fomentations to the bowels, and sometimes turpentine epithems. When there is a tendency to convalescence, or the system seems to be sinking, I administer wine, sul. cinchonia, pulv. camphor and carb. ammonia: or rather select out of those articles such as I think are most demanded. My prescriptions during the fall under such circumstances, constituted of sul. cinchonia grs. iii, camphor grs. iv, carb. ammo. grs. iii, mix, and give every four hours, and alternate with wine from 2 to 3 teaspoonfuls.

Would say, I had no confidence in the quinia in the first stages of this disease; believe it really to be detrimental. In conclusion, I would add, that the above is my treatment in the well marked cases; complicated ones, of course, require variation in treatment.

Cholera Infantum has not prevailed with us during the year. A few isolated cases made their appearance. My treatment is principally minute doses of calomel until the liver is sufficiently acted upon; then actate plumbi combined with minute doses of ipecac, and sometimes creta prep. or lime water, administered at the same time.

*Stomatis Materna* has never prevailed here as an epidemic. I occasionally see an isolated case of it.

In concluding this article in reply to your interrogatories, I would state, that during the time that erysipelas was prevailing in our community, several women were confined under my care; and two out of this number were attacked with puerperal peritonitis, both of whom died. I would call the attention of the profession to this subject as another proof of the strong similarity, if not identity, of the two diseases,—showing conclusively, that when one is an epidemic the other is also; and that when one prevails, the other does likewise.

In reply to query No. 8, I would state, that in former years I had but little confidence in any medication in scarlatina; but a year or two since I saw an article from a French physician, recommending the free administration of carb. ammo. in this disease, stating that out of 50 or 60 patients, none died. I resolved at once to give this article a trial on the first cases that should present themselves to me. Very soon I had an opportunity in some malignant cases connected with spasms, &c. One little patient in particular, I thought would certainly die. I immediately made a solution, so that each dose would contain as much as 3 grains, and ordered it to my little patient every hour or two. I had the satisfaction to see a complete recovery, and all the patients, amounting to 13 or 14 in the neighborhood, were treated in this *heroic* treatment, and with complete success. I have the confidence under similar circumstances with the remedy, to use it in the same way, should an epidemic occur in our midst.

All of which is respectfully submitted,

HIRAM NANCE, M.D.

---

The following is from Dr. Haller, of Vandalia:—

C. Goodbrake, M. D.,

*Dear Sir:*—As usual, the prevailing diseases have been malarial, with a greater degree of congestion and nervous inervation than ordinarily. The stomach and bowels being so irritable from congestion, as to prevent the early administration of antiperiodics; consequently many of them assumed a continued form, and for days would resist all our efforts to bring them to a favorable issue. Calomel in small doses, often repeated with sulph. morphia and bi-carbonate of soda, I found the most efficient agents in these cases, while the irritability of the stomach and fever continued: after they would subside, I usually gave a few sedative doses of quinine, which this year was about 8 grs., when formerly it took some 12 or 15 to have the same effect. For some time past I have observed that patients required much less quinineism than formerly. I am unable to account for this change.

As winter approached, these symptoms became more mild; the cases that occurred up to February were, as a general thing, of a mild type. Now pneumonia, accompanied with severe functional diseases of the billiary organs and great nervous debility, set in; several cases I had, proved fatal; the complications were of such gravity as to show but too surely the grave claimed another victim. These cases all required the tonic and supporting treatment from the beginning; indeed, any other treatment would have hastened them on to a dissolution. Many were under this course from the excessive nervous debility, commence sweating the cadaverous sweat, the countenance becoming hippocratic, they sinking with all the appearance of having died of cholera or pernicious fever. The only means I found of availability, was quinine with diffusible stimulants and extensive vessication in the early stage of the attack, and if I could bring my patients thoroughly under these effects before the collapsed stage I could save them; but if this took place, or if the disease had been permitted to run so long, they invariably died. The symptoms in these cases were the most grave I have witnessed for years; occasionally we have an apparent epidemic of this kind, with the symptoms and course of the disease as follows: The lungs are first attacked with congestion, or rather engorgement; then deep seated congestion of the liver, stomach, spleen, kidneys, spine, and brain, finally ends the drama; the patient soon succumbing from deep congestion of all the vital organs. Some die in a few hours; others by reason of great strength, last two or three days, during which time there is no reaction: these cases have always proved fatal in my hands, and on inquiry, I learn others are just as unsuccessful. In conversation with Prof. John S. Moore, of St. Louis, in relation to such cases, he informed me that when he practised at Carlisle, on the Kaskaskia River, he occasionally had such cases; saved but one, and her recovery he attributed to early blistering her entire spine, with the free use of calomel, quinine and stimulants, which he thought was the only treatment that would be likely to effect anything toward a cure. Encouraged by his success, I tried these agents effectually; but they proved abortive in my hands. These cases continued to occur up to the middle of April, since which time diseases have been of a mild type. Notwithstanding the prevalence of these grave cases, the last year has been one of universal good health.

Diseases are undergoing changes continually. The intermittents and remittents that used to be so rife in this section, are becoming less frequent, and those of a continued type of more usual occurrence. Diseases of the respiratory organs are increasing; also nervous diseases are becoming quite prevalent: consequently the treatment has changed,—the old routine practice, with intelligent physicians, has become obsolete —less of the heroic and more of the expectant plan is adopted, allowing our patients as much nutritious diet as the digestive organs can manage; or in other words, we support the powers of nature while she effects the cure, instead of the abortive treatment we find so efficient in our bilious intermittents and remittents. Diseases have so changed as to generally require the expectant and supporting plan of treatment rather than the abortive.

This section has been exempt from typhoid fever the last year; it is a disease that seldom occurs here—only now and then a case occurring: therefore, I have formed no new ideas of its pathology or treatment from that laid down by our standard authors. I treat the cases I have on general principles, with satisfactory results. I have no new suggestions to offer in the treatment of disease that would be of any practical advantage.

I have before given the topography of our county.

Yours respectfully,

F. B. HALLER.

---

We have had but very little sickness in De Witt County during the last year. Through the autumnal months we had some cases of remittent fever which proved unusually stubborn —and several deaths occurred. The administration of quinine was not attended with any benefit whatever, and the old plan of giving calomel and diaphoretics with effervessing draughts, seemed to be attended with the best results.

During the winter we were visited with an epidemic of hooping-cough which carried off a number of children. In all the fatal cases—so far as I know—the brain became affected. The little patient would become comatose, and in from twelve to twenty-four hours from the time these symptoms made their appearance, convulsions would set in, which soon terminated in death. The treatment which seemed to answer the best purpose, was occasional small doses of calomel, and oil or enemas to open the bowels when necessary; when the brain became implicated, enemas served the best purpose; and in those cases where convulsions were threatened, blisters were applied to the back of the neck.

The following mixture seemed to answer a very good purpose throughout the continuance of the disease:

| | | |
|---|---|---|
| ℞ | Pulv. Coccinellae, | ℈ ss. |
| | Carb. Potassa, | ℈ i. |
| | Sacchar. Alba, | ʒ ii. |
| | Fl'd Ext. Asclip Tuber, | ℥ i. |
| | Aqua. Destil, | ℥ iv. |

Misce, S. Give a teaspoonful—to a child—every three or four hours, or, according to indications. I believe the mixture will —in ordinary cases—cut the disease short; and in protracted cases it will alleviate the most distressing symptoms of the disease. Occasionally assafœtida was given with good effect. But the most troublesome disease we have had to deal with in this vicinity for the last two years, is diphtheria. This complicated and fatal disease made its appearance in our County in September 1858, since which time it has proved fatal in a great many cases—the attacks being about in the proportion of two adults to three children.

There has been so much said and written upon this subject lately, that it is unnecessary to enter into a description of the symptoms, and I will only state that the trouble about the fauces and tonsils was not so difficult to get along with as the general prostration of the system; and other symptoms which would in almost all cases supervene throughout the course of the disease. Among these symptoms, I may mention the irregular action and painfulness of the muscles of the neck, swelling, and sometimes suppuration of the parotid gland, pain, and sometimes convulsive twichings of the muscles of the extremities.

As a local application to the fauces and tonsils, we found the mur. tr. iron to answer the best purpose in all cases that came under our treatment. We tried the nitrate of silver, sulp. of copper, alum, and alum and sulp. of copper combined ; but the tinct. of iron seemed to have the best effect in our hands.

The best remedies internally, seemed to be quinine, tinct. of iron, chlorate of potassa, and good porter or brandy with good nutritive diet.

